# EHMTI-0369. Migraine is a dysfunction of the mind – in networks creating reality

**DOI:** 10.1186/1129-2377-15-S1-L1

**Published:** 2014-09-18

**Authors:** K Hougaard, E Zunthe

**Affiliations:** 1 Neurology, Pun Hlaing International hospital, Yangon, Myanmar, Burma

## Introduction

Thinking of migraine pathophysiology is today constrained by a post Wolff intellectual fixation with pain as the main migraine hallmark, and constrained by the rigid syndrome classification which excludes the understanding of other sufferings from the same dysfunction contributes to the creation of concepts. That fixedness is a significant obstacle to understand migraine as a general dysfunction of the brain.

## Method

Knowledge of how the brain works on the system level has developed within the field of cognitive neuroscience. This knowledge is built on what brains does: It register the world, creates an understanding of reality, and acts according to goals. This approach is somewhat different from the classic neuron-approach of neurology, looking at what single neurons does.

We apply knowledge from cognitive neuroscience to present an understanding of what migraine is.

## Result

Migraine is a dysfunction in network creation of what Bernard Baars describes as 'context' or 'frame'. Stanislas Dehaene is calling the same unconscious ongoing creation of a coherent workable reality 'precociousness'. This function is reflected in the P300 potentials.

## Conclusion

We suggest a form to build knowledge about migraine brain dysfunction. We believe Albert Einstein knew an absolute and basic truth of how the human brain creates knowledge:

'It seems that the human mind has first to construct form independently before we can find them in things....the truth [is] that knowledge cannot spring from experience alone, but only from a comparison of the inventions of the intellect with observed facts.

A. Einstein 1930

No conflict of interest.

